# Exploring pharmacological strategies in the management of ARDS: Efficacy, limitations, and future directions

**DOI:** 10.2478/jccm-2025-0030

**Published:** 2025-07-31

**Authors:** Sultan Almuntashiri

**Affiliations:** Department of Clinical Pharmacy, College of Pharmacy, University of Ha’il, Ha’il, Saudi Arabia

**Keywords:** ARDS, acute lung injury, beta agonists, NMBAs, corticosteroids

## Abstract

Acute respiratory distress syndrome (ARDS) is a severe inflammatory reaction in the lungs caused by sudden pulmonary and systemic injuries. Clinically, this diverse syndrome is marked by sudden hypoxemic respiratory failure and the presence of bilateral lung infiltrates visible on a chest X-ray. ARDS management remains largely supportive, with a focus on optimizing mechanical ventilation strategies and addressing the underlying causes of lung injury. The current pharmacological approach for ARDS primarily focuses on corticosteroids, neuromuscular blocking agents, and beta-2 agonists, however, none has been definitively proven to be consistently effective in improving clinical outcomes. This review summarizes the latest evidence regarding the effectiveness and limitations of these pharmacological interventions, identifying key areas where further research is needed.

## Introduction

Acute respiratory distress syndrome (ARDS) is a severe inflammatory reaction in the lungs caused by sudden pulmonary and systemic injuries [1]. In the pulmonary microvasculature, severe lung inflammation leads to injury of the alveolar epithelial cells and the capillary endothelial cells [2]. Clinically, this diverse syndrome is marked by sudden hypoxemic respiratory failure and the presence of bilateral lung infiltrates visible on a chest X-ray. Most patients with ARDS require sedation, intubation, and mechanical ventilation, along with pharmacologic interventions aimed at treating the underlying cause and managing ARDS-related complications [3].

ARDS management remains largely supportive, with a focus on optimizing mechanical ventilation strategies and addressing the underlying causes of lung injury. The current pharmacological approach for ARDS primarily focuses on corticosteroids, neuromuscular blocking agents (NMBAs), and beta-2 agonists; however, none has been definitively shown to improve outcomes consistently [4]. This review summarizes current evidence on the therapeutic use of these agents, comparing findings across major trials, discussing their clinical impact and limitations, and identifying key areas where further research is needed.

## Search strategy and study selection

**Fig. 1.**
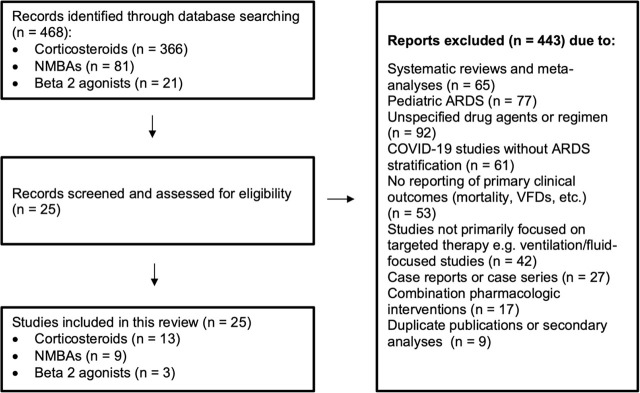
Search strategy and study selection

We searched PubMed, MEDLINE, and ClinicalTrials.gov for clinical studies using the terms: *“acute respiratory distress syndrome” OR “ARDS”* combined with *“corticosteroids”*, *“neuromuscular blocking agents”*, or *“beta-2 agonists”*. We included randomized controlled trials and cohort studies reporting key clinical outcomes (e.g., mortality, ventilator-free days, ICU stay, or adverse events). Studies in pediatric populations or without relevant pharmacologic interventions were excluded. The study selection process is illustrated in Figure 1.

## Critical Appraisal of Key Studies

To provide a foundation for this review, Table 1 presents a critical appraisal of major studies evaluating pharmacologic interventions in ARDS. These studies were selected based on their clinical relevance, methodological quality, and influence on current treatment practices. The table offers a structured overview, summarizing study designs, potential sources of bias, and the strength of supporting evidence.

**Table 1.** Appraisal of Key Clinical Studies of Pharmacologic Agents in ARDS

**Reference**	**Drug Class**	**Study Design & Setting**	**Bias Risk**	**Evidence Strength**
Steinberg et al. [5]	Corticosteroids	Multicenter RCT; persistent ARDS	Well-designed-blinded but late intervention	Moderate
Meduri et al. [6]	Corticosteroids	Multicenter RCT; early severe ARDS	Well-designed-blinded and early intervention; limited sample size	Moderate
Meduri et al. [7]	Corticosteroids	Multicenter RCT; prolonged ARDS	Blinded, limited sample size	Moderate
Villar et al. [8]	Corticosteroids	Multicenter, randomized, double-blind RCT	Strong methodological design with blinding	High
Tomazini et al. [9]	Corticosteroids	Multicenter, randomized, open-label controlled trial	Lack of blinding	Moderate
Papazian et al. [10]	NMBAs	Multicenter, randomized, double-blind RCT	Strong design with blinding and protocol standardization	High
Moss et al. [11]	NMBAs	Multicenter, randomized, open-label controlled trial	Lack of blinding; early termination for futility	Moderate
Forel et al. [12]	NMBAs	Multiple-center, prospective, controlled trial	Well-designed with blinding; limited by small sample size	Moderate
Gainnier et al. [13]	NMBAs	Multiple center, prospective, controlled trial	Strong blinding and randomization; small sample size	Moderate
Perkins et al. [14]	Beta-2 agonists	Single-center, randomized, double-blind placebo-controlled trial	Well-designed with blinding; small sample size limits generalizability	Moderate
Matthay et al. [15]	Beta-2 agonists	Multicenter, randomized, double-blind placebo-controlled trial	Strong design with adequate blinding; some heterogeneity in patient severity	Moderate
Gao Smith et al. [16]	Beta-2 agonists	Multicenter, randomized, double-blind, placebo-controlled trial	High-quality design with rigorous blinding; stopped early for safety concerns	Moderate

Randomized Clinical Trial (RCT); Neuromuscular blocking agents (NMBAs); Acute respiratory distress syndrome (ARDS)

## Pharmacological interventions for ARDS

### Corticosteroids

There has been significant interest in the use of corticosteroids to reduce pulmonary and systemic damage in ARDS patients due to their strong anti-inflammatory and antifibrotic effects. Corticosteroids are recommended for patients suffering from pneumonia, sepsis, and septic shock, as these conditions can lead to ARDS [17]. Administering low-dose corticosteroids may reduce the transcription of proinflammatory cytokines, helping to prevent a prolonged cytokine response and potentially speeding up the resolution of systemic and lung inflammation in the early stages of ARDS [17, 18]. While corticosteroids are commonly used in the management of ARDS, key aspects such as the optimal agent, dosing strategy, and treatment duration remain uncertain.

Corticosteroids act as agonists at either glucocorticoid receptors or mineralocorticoid receptors. Glucocorticoid receptors mediate the anti-inflammatory effects and hyperglycemia while mineralocorticoid receptors influence the renin-angiotensin-aldosterone system, resulting in sodium retention, which can lead to volume retention and hypernatremia [19]. Dexamethasone may offer the beneficial anti-inflammatory activates without causing mineralocorticoid stimulation, which can lead to sodium and fluid retention that may worsen lung injury [20]. However, hydrocortisone has the same affinity for both glucocorticoid and mineralocorticoid receptors. The mineralocorticoid properties of hydrocortisone may be advantageous in cases of vasodilatory shock for maintaining intravascular volume, but they may be undesirable for patients with ARDS [21]. In addition, dexamethasone is suitable for a once-daily dosing regimen because of its long-lasting pharmacological effects. In *coronavirus disease 2019 (COVID-19*), dexamethasone is recommended as the first corticosteroid to be used, while other corticosteroids like hydrocortisone and methylprednisolone are considered as alternatives at equivalent doses [22]. Therefore, early adjunctive treatment with intravenous dexamethasone, alongside standard supportive care, may help reduce pulmonary and systemic inflammation in patients with moderate-to-severe ARDS, potentially shortening the duration of mechanical ventilation and reducing mortality.

Ventilator-free days (VFDs) are frequently used as a composite outcome measurement or the primary outcome in ARDS clinical trials [23]. Other outcomes like intensive care unit (ICU) length of stays, hospital length of stays and mortality are commonly measured as secondary outcomes [24]. In two *randomized controlled trials* (*RCTs*), moderate-to-severe ARDS patients treated with systemic dexamethasone showed significant improvements in the VFDs than control group [8,9]. For the death outcome, Villar J et al reported that dexamethasone significantly reduced the mortality rate at 60 days [8] while other studies did not show similar observations [9,25,26]. Comparing to other corticosteroids, dexamethasone group exhibited a lower overall mortality rate during the 28-day follow-up period when compared to methylprednisolone and hydrocortisone groups, but this percentage did not achieve a statistically significant level [25]. Other outcomes like ICU-free days, length of ICU stays, and length of hospital stays were not significantly improved after dexamethasone treatments [9, 25]. (Table 2)

**Table 2.** Corticosteroid in ARDS

**Reference**	**Study design**	**Number of patients**	**Corticosteroid type and dose**	**Key findings**
[5]	RCT	180 patients with ARDS Methylprednisolone (n=89) Placebo (n=91)	Methylprednisolone 2 mg/kg Followed by 0.5 mg/kg q 6 h for 14 days Followed by 0.5 mg/kg q 12 h for 7 days, and then tapering the dose over 2 or 4 days	↑ VFDs (11.2 vs 6.8 days; *P*<0.001) at day 28 ↑ ICU free days (8.9 vs 6.2 days; *P*=0.02) at day 28 ↑ VFDs (159 vs 149 days; *P*=0.04) at day 180 ↔ ICU free days at day 180 ↔ 60- or180-day mortality
[6]	RCT	91 patients with severe early ARDS Methylprednisolone (n = 63) Placebo (n = 28)	Methylprednisolone infusion (1 mg/kg/d) for up to 28 days	↑ VFDs (16.5 vs 8.7 days; *P*=0.001) at day 28 ↑ PaO_2_/FiO_2_ ratio (256 vs 179; *P* = 0.006) on day 7 ↓ Length of ICU stay (7 vs 14.5 days; *P* = 0.007) at day 28 ↓ ICU mortality (20.6 vs 42.9%; *P* = 0.03) at day 28 ↔ Length of hospital stay at day 28
[31]	RCT	216 patients with COVID-19 induced-ARDS Dexamethasone group (n=111) Methylprednisolone (n=105)	IV dexamethasone 6 mg daily for 7 to 10 days Methylprednisolone 250 – 500 mg daily for 3 days followed by oral prednisone 50 mg daily for 14 days	↓ Mortality (7.4 vs 36.9 %; *P*< 0.0001) at day 30 for methylprednisolone than dexamethasone ↑ Recovery time (3 vs 6 days; *P* < 0.0001) for methylprednisolone than dexamethasone
[7]	RCT	24 patients with severe ARDS Methylprednisolone (n=16) Placebo (n=8)	Methylprednisolone 2 mg/kg per day **→ taper over 32 days** Loading: 2 mg/kg IV once Days 1–14: 2 mg/kg/day Days 15–21: 1 mg/kg/day Days 22–28: 0.5 mg/kg/day Days 29–30: 0.25 mg/kg/day Days 31–32: 0.125 mg/kg/day	↓ Mechanical ventilation duration (11.5 vs 23 days; *P* = 0.001) ↓ ICU mortality (0 vs 62%; *P*=0.002) ↓ Hospital mortality (12 vs 62%; *P*=0.03) ↓ Lung injury score (LIS) (1.7 vs 3.0; *P*<0.001) ↑ PaO_2_/FiO_2_ ratio (262 vs 148; *P*<0.001) ↑ Successful extubation (7 vs 0; *P*=0.05) ↑ Organ failure free days (16 vs 6 days; *P* =0.005)
[32]	RCT	81 Mechanically ventilated patients at high risk for ARDS Methylprednisolone (n=39) Placebo (n= 42)	Methylprednisolone 30 mg/kg, q 6 hours for 48 hours	↔ Oxygen requirements ↔ Days of intensive care ↑ Incidence of infection rate (*P* < 0.05)
[29]	Cohort	20 postoperative ARDS patients Placebo (n=8) Methylprednisolone (n=12)	Loading dose 2 mg/kg followed by 2 mg/kg/day IV daily q 6 hours and then changed to a single oral dose or discontinued	↓ Mortality (8.3 vs 87.5%; *P*= 0.001) ↑ PaO_2_/FiO_2_ ratio on day 4 (*P* < 0.05)
[36]	RCT	99 ARDS patients Methylprednisolone (n=50) Placebo (n=49)	Methylprednisolone (30 mg/kg q 6 hours for 24 hours)	↔ Mortality at 45 days
[30]	Secondary analysis of RCT	745 ARDS patients No corticosteroids (n=506) Corticosteroids (n=239)	IV or PO corticosteroids (e.g. methylprednisolone /dexamethasone/prednisone/hydrocortisone) **Doses**: 20 mg methylprednisolone / 3.75 mg dexamethasone/ 25 mg prednisone/ 100 mg hydrocortisone daily	↔ Mortality
[8]	RCT	277 patients with moderate-to-severe ARDS Dexamethasone group (n=139) Control group (n=138)	Dexamethasone IV 20 mg once daily for 5 days and then reduced to 10 mg daily from day 6 to day 10	↑ VFDs (12.3 vs 7.5 days; *P*<0.0001) at 28 days ↑ PaO_2_/FiO_2_ (*P* < 0.05) on day 6 ↓ Mortality at 60 days (21% vs 36%, *P* = 0.0047) ↓ SOFA score on day 3 (*P* < 0.05) ↔ Adverse events
[9]	RCT	299 patients with COVID-19–associated with moderate to severe ARDS Dexamethasone (n =151) or Control (n = 148)	Dexamethasone IV 20 mg once daily for 5 days and then reduced to 10 mg for additional 5 days or until ICU discharge	↑ VFDs (6.6 vs 4 days; *P* = 0.04) at 28 days ↓ SOFA (6.1 vs 7.5; *P* = 0.004) at 7 days ↔ Mortality at 28 days ↔ ICU-free days at 28 days
[25]	RCT	106 patients with mild to moderate COVID-19-related ARDS Methylprednisolone (n=36), Dexamethasone (n=35), Hydrocortisone (n=35)	Dexamethasone IV 6 mg once daily for 10 days Methylprednisolone IV 16 mg BID for 10 days Hydrocortisone IV 50 mg TID for 10 days	↔ VFDs ↔ PaO_2_/FiO_2_ ratio ↔ ICU stays ↔ Hospital stays ↔ 28-day mortality ↔ Adverse events
[26]	RCT	98 patients with COVID-19-related ARDS High dexamethasone groups (n=49) Low dexamethasone groups (n=49)	16 mg of dexamethasone IV daily for 5 days followed by 8 mg for 5 days or 6 mg of dexamethasone IV daily for 10 days.	↔ VFD between high- and low-dose dexamethasone groups at 28 days ↑ **Successful extubation** on high dose group (*P* < 0.05) ↔ Adverse events
[37]	RCT	197 patients with sepsis related ARDS Hydrocortisone (n = 98) Placebo (n = 99)	Hydrocortisone IV 50 mg q 6 h daily for 7 days	↑ PaO_2_/FiO_2_ (319.1 vs 266.3; **P** = 0.001) ↔ Mechanical ventilation duration at day 28 ↔ Mortality at 28 days

Randomized Clinical Trial (RCT); Partial pressure of oxygen in arterial blood (PaO_2_) to the fraction of inspiratory oxygen concentration (FiO_2_); Ventilator free days (VFDs); Intensive care unit (ICU); Sequential Organ Failure Assessment (*SOFA*); Multiple organ dysfunction syndrome (MODS); Lung Injury *Score (*LIS); ↑ = Increase; ↓= Decrease; ↔ = No significant change

The ratio of the partial pressure of oxygen in arterial blood (PaO_2_) to the fraction of inspired oxygen (FiO_2_) is a crucial component in evaluating patients with ARDS. Based on the PaO_2_/FiO_2_ results, ARDS patients can be categorized into three groups either mild, moderate, or severe [27]. In one study, the dexamethasone group demonstrated a notable improvement in the PaO_2_/FiO_2_ ratio, suggesting its potential benefit in enhancing oxygenation in ARDS patients [8]. However, conflicting findings were reported in another study, where dexamethasone administration did not result in any significant improvement in the PaO_2_/FiO_2_ ratio [25]. Given these inconsistent results, it becomes crucial to explore the underlying mechanisms driving these differences. Further studies are needed to comprehensively assess the efficacy of dexamethasone in improving oxygenation in ARDS patients. Additionally, research should aim to identify specific subgroups of ARDS patients who might derive the most benefit from dexamethasone therapy, considering factors such as the timing, dosage, and duration of administration.

The Sequential Organ Failure Assessment (SOFA) score was created to offer an easy way to evaluate and monitor organ dysfunction in critically ill patients. It is a scoring system that evaluates the functioning of various organ systems in the body, including neurological, hematologic, hepatic, renal, and hemodynamic parameters, and assigns a score based on the information gathered in each category. A higher SOFA score indicates a greater likelihood of mortality [28]. In two independent clinical trials, patients treated with dexamethasone showed less SOFA score in comparison to patients received other therapies for managing ARDS [8,9].

In ARDS study included 180 patients, methylprednisolone significantly increased the number of VFDs at day 28 and at day 180 [5]. Similarly, methylprednisolone treatment was associated with an improvement in the mechanical ventilation-free days at day 28 in patients with severe early ARDS [6]. In a study included patients with unresolving severe ARDS by the seventh day, methylprednisolone also showed improvements in the outcome of duration of mechanical ventilation [7]. These studies advocate for the use of methylprednisolone in the treatment of ARDS, irrespective of whether the disease is in its early or late stages.

In patients with severe early ARDS, methylprednisolone treatment was associated with an improvement in ICU mortality [6]. Similarly, early dose of methylprednisolone showed significant improvement in mortality in postoperative ARDS patients [29]. Methylprednisolone also showed significant improvements in mortality outcome in patients with unresolving or late ARDS [7]. In a secondary analysis of Statins for Acutely Injured Lungs from Sepsis (SAILS) trial, corticosteroids showed no significant effect on death after adjustment for other potential confounders using regression analysis approach [30]. In a study compared different corticosteroids, methylprednisolone showed less mortality than dexamethasone at 30-day follow-up [31]. Regarding other clinical outcomes, methylprednisolone treatment was associated with an improvement in the length of ICU stay in a study included patients with severe early ARDS [6] while another study included ventilated patients at high risk for ARDS showed no significant differences in days of intensive care after methylprednisolone treatment [32]. This discrepancy might be due to differences between the groups, as the latter study included patients at high risk for ARDS.

There is a significant debate in the literature regarding the timing of corticosteroid treatment for ARDS, whether administered early or late. In one study, initiating methylprednisolone treatment more than two weeks after ARDS begins raised the risk of death [5] while another study stated that prolonged use of methylprednisolone in patients with persistent ARDS was linked to improvements in lung injury and reduced the mortality rates [7]. Likewise, in patients with early severe ARDS (less than 72 hours), methylprednisolone treatment was associated with an improvement in the mechanical ventilation-free days at day 28, length of ICU stay, and ICU mortality [6]. Administering low-dose methylprednisolone in the early stages of ARDS showed also significant effects on reducing mortality [29]. In experimental acute lung injury (ALI) model, early methylprednisolone treatment preserved both in vivo and in vitro respiratory mechanics in mild lung injury, and in severe ALI, it minimized alterations in tissue impedance and the extracellular matrix [33]. Prolonged glucocorticoid administration in experimental studies showed positive effective in reducing lung collagen and edema formation, while stopping the drug quickly reversed these beneficial effects [34,35]. These studies offered mechanistic insights into the advantages of early and extended administration of glucocorticoids.

## Neuromuscular blocking agents

Neuromuscular blocking agents (NMBAs) induce skeletal muscle paralysis by blocking nerve signal transmission at the neuromuscular junction, making them useful in the ICU for facilitating endotracheal intubation in patients with reduced lung compliance like ARDS [38]. The most common reason for using neuromuscular blockade in ARDS patients is the difficulty in mechanically ventilating the patient due to unusually high airway pressures, abnormal ventilation rates, or improper cycling times. Therefore, NMBAs could be helpful in lung-protective ventilation by reducing patient–ventilator asynchrony, decreasing the work of breathing, and limiting alveolar fluid buildup, all of which could be beneficial for ARDS patients [38,39,40]. They are also believed to have anti-inflammatory effects, reducing downstream cytokine release by inducing paralysis and preventing lung and systemic organ damage associated with ARDS and mechanical ventilation [41].

Neuromuscular blocking agents are classified into two primary groups: depolarizing and nondepolarizing. Succinylcholine, a depolarizing NMBA, is commonly used in procedural settings like rapid sequence intubation due to its pharmacokinetic properties. Nondepolarizing agents are categorized into two groups based on their chemical composition: steroidal (e.g., rocuronium, vecuronium, pancuronium) and benzylisoquinoline (e.g., mivacurium, atracurium, cisatracurium). These agents act as competitive antagonists to acetylcholine (ACh) by binding to nicotinic receptors on the postsynaptic membrane, leading to muscle paralysis [38].

In ARDS, cisatracurium is the preferred neuromuscular blocking agent due to its favorable safety profile. It is less likely to cause myopathy, does not induce histamine release, has a short half-life, and is eliminated through organ-independent pathways, avoiding reliance on liver or kidney function [42]. While other agents, such as atracurium and vecuronium, have also been evaluated in ARDS, their use has been investigated in far fewer clinical studies compared to cisatracurium.

The ARDS et Curarisation Systematique (ACURASYS) trial was a multicenter, double-blind trial included 340 patients with moderate to severe ARDS (PaO_2_/FiO_2_ < 150 mm Hg) within 48 hours of ICU admission and the subjects were randomized to receive either 48 hours of cisatracurium or placebo. This study showed lower mortality risk, more VFDs and more ICU free days without notable adverse events in patients received cisatracurium than a usual care with a strategy of deep sedation and without routine neuromuscular blockade [10]. (Table 3)

**Table 3.** Neuromuscular blocking agents in ARDS

**Reference**	**Study design**	**Number of patients**	**NMBAs type and dose**	**Key findings**
[10]	RCT	339 patients with moderate to severe ARDS Cisatracurium (n=177) Placebo (n=162)	15 mg of cisatracurium followed by a continuous infusion of 37.5 mg/hour for 48 hours	↓ Hazard ratio of mortality. HR= 0.68 (95% CI, 0.48 to 0.98; P = 0.04) ↓ Mortality (23.7 vs 33.3 %; P=0.05) at 28-day ↓ Mortality (30.8% vs 44.6%, P=0.04) in patients with baseline PaO_2_/FiO_2_ <120 mm Hg ↔ Mortality at 90-day ↑ VFDs (10.6 vs 8.5 days; P=0.04) at 28 days ↑ VFDs (53.1 vs 44.6 days; P=0.03) at 90 days ↑ ICU free days (47.7 vs 39.5; P=0.03) at 90 days ↔ ICU free days at 28 days
[11]	RCT	1006 patients with moderate-severe ARDS Cisatracurium group (n=501) Control group (n=505)	15 mg of cisatracurium followed by a continuous infusion of 37.5 mg/hour for 48 hours	↔ 90 days mortality ↔ Hospital death at 28 days ↔ VFDs at 28 days ↔ ICU free days at 28 days ↔ Hospital free days at 28 days ↑ Cardiovascular adverse events (14 vs 4 events; P=0.02)
[12]	RCT	36 patients with moderate-severe ARDS Cisatracurium (n = 18) Placebo (n = 18)	A bolus dose of cisatracurium 0.2 mg/kg was followed by a continuous infusion at an initial rate of 5 μg/kg/min for 48 hr	↔ Mechanical ventilation duration ↔ VFDs at 28 days ↔ ICU mortality ↑ PaO_2_/FiO_2_ ratio (P < 0.001)
[13]	RCT	56 patients with moderate-severe ARDS Cisatracurium (n=28) Placebo (n=28)	50 mg bolus of cisatracurium followed by a continuous infusion at an initial rate of 5 μg/kg/min for 48 hr	↑ PaO_2_/FiO_2_ ratios (P < 0.05) ↔ ICU mortality ↔ VFDs at day 28 ↔ VFDs at day 60
[47]	Retrospective cohort study	172 patients with moderate-severe ARDS Control (n = 86) Cisatracurium (n = 86)	NA	↓ Length of ICU stay (9.37 vs 14.67 days; P <0.01) ↓ Duration of ventilation (6.40 vs 12.38 days; P <0.01) ↔ Length of hospital stay ↔ Mortality outcomes at 28-day, 90-day, or 1-year
[44]	Retrospective cohort study	58 patients with moderate-severe ARDS Cisatracurium (n=29) Vecuronium (n=29)	The treatment duration was around three days (74.2 vs 69.6 hr) in cisatracurium and vecuronium, respectively The average daily dose 123.7 vs 63 mg/day in cisatracurium and vecuronium, respectively The hourly infusion rate 7.9 vs 4.4 mg/hr in cisatracurium and vecuronium, respectively	↔ Ventilator days ↔ ICU mortality ↔ Hospital mortality ↔ Length of ICU stay ↔ Hospital length of stay ↔ PaO_2_/FiO_2_ ratio after 48 hours
[43]	Retrospective cohort study	3802 patients with ARDS or at risk for ARDS Cisatracurium (n=1,901) Vecuronium (n=1,901)	Continuous infusion of NMBA for at least 2 days. The exact dose is not available.	↔ Mortality outcome ↔ Hospital length of stay ↓ Ventilator days (P = 0.005) in patients treated with cisatracurium ↓ ICU length of stay (P = 0.028) in patients treated with cisatracurium
[45]	Retrospective cohort study	76 patients with severe ARDS Atracurium (n=18) Cisatracurium (n=58)	The treatment duration (2.5 vs 2.6 days) in atracurium and cisatracurium, respectively The treatment dose (1.9 vs 2.5 μg/kg/min) in atracurium and cisatracurium, respectively	↔ PaO_2_/FiO_2_ at 72 hr ↔ VFDs at day 28 ↔ ICU length or stay ↔ Hospital length of stay ↔ Hospital mortality
[46]	Retrospective cohort study	225 patients with ARDS Atracurium (n = 75) Cisatracurium (n = 150)	Atracurium 4 µg/kg/min, titrated by 0.5 µg/kg/min q 15 minutes to a maximum dose of 20 µg/kg/min for at least 12 hr Cisatracurium 1 µg/kg/min, titrated by 0.5 µg/kg/min q 15 minutes to a maximum dose of 10 µg/kg/min for at least 12 hr	↑ PaO_2_/FiO_2_ ratio (68.6 vs 54.6; P= 0.011) for atracurium than cisatracurium at 24 hours, ↑ PaO_2_/FiO_2_ ratio (52.3 vs 41.3; P=0.014) for atracurium than cisatracurium at 72 hours ↔ PaO_2_/FiO_2_ ratio at 48 hours ↔ ICU length of stay ↔ Hospital length of stay ↔ Duration of mechanical ventilation ↑ Hospital mortality (58.7 vs 36 %; P=0.001) in atracurium than cisatracurium

Randomized Clinical Trial (RCT); Partial pressure of oxygen in arterial blood (PaO_2_) to the fraction of inspiratory oxygen concentration (FiO_2_); *Hazard ratio (HR)*; Simplified Acute Physiology Score II (SAPS II); Ventilator free days (VFDs); Intensive care unit (ICU); Not available (NA); Neuromuscular blockade agents (NMBAs); ↑ = Increase; ↓= Decrease; ↔ = No significant change

The Re-evaluation of Systemic Early Neuromuscular Blockade (ROSE) trial focused on evaluating the effectiveness and safety of initiating NMBA early versus standard care using light sedation approaches among moderate to severe ARDS patients. The patients were assigned to either 48-hour continuous infusion of cisatracurium with deep sedation approach or usual care with lighter sedation strategy [11]. In patients with moderate-to-severe ARDS, the ROSE trial found no significant difference in 90-day mortality between those receiving early, continuous cisatracurium infusion and those managed with standard care and lighter sedation targets. The trial was terminated early due to futility. In addition, no significant differences between the groups in secondary end points at 28 days including hospital death, VFD, ICU free days and hospital free days. However, more cardiovascular adverse events were seen in patients received cisatracurium. It is speculated that deep sedation in patients receiving cisatracurium might be a potential reason for the higher percentage of cardiovascular adverse events [11].

The ROSE trial was designed to align with specific aspects of the ACURASYS study. Similarities included are the utilization of the same neuromuscular blocking agent, cisatracurium, following a similar dosing regimen and treatment duration. One of the most striking contrasts observed across the studies lies in the varied methodologies employed, particularly in sedation approaches, positive end-expiratory pressure (PEEP) strategies, and the use of prone positioning. ACURASYS applied deep sedation approach in both the intervention and control groups, whereas in the ROSE trial the deep sedation was only utilized in the intervention group. Higher PEEP (≥8 cm H_2_O) was applied in ROSE as higher PEEP itself may affect clinical outcomes among moderate-to-severe ARDS patients while ACURASYS trial utilized a low PEEP strategy of ≥5 cm H_2_O. In addition, the number of patients who underwent prone positioning in ROSE trial was lower than in the ACURASYS trial. Prone positioning is believed to enhance oxygenation by minimizing the pleural pressure gradient through gravitational effects. Other key differences included the number of patients enrolled, the time from ARDS diagnosis to inclusion, and the lack of blinding in the ROSE trial, which may have influenced the assessments of therapies. Altogether, these key differences could explain the inconsistencies in the results of the ACURASYS and ROSE trials.

A multicenter, prospective, controlled, and randomized trial involving 36 patients with ARDS (characterized by a PaO_2_/FiO_2_ ratio ≤200 and a PEEP ≥5 cm H_2_O) within 48 hours of onset demonstrated that treatment with cisatracurium significantly improved the PaO_2_/FiO_2_ ratio over a 120-hour study period [12]. Similarly, another multicenter, prospective, randomized, and controlled trial, which included 56 patients with ARDS (defined by a PaO_2_/FiO_2_ ratio <150 and a a PEEP ≥5 cm H_2_O), found that the cisatracurium group exhibited higher PaO_2_/FiO_2_ ratios at various time points, including 120 hours, compared to the placebo group [13]. However, despite these improvements, neither trial demonstrated a statistically significant difference between the groups in terms of VFDs at 28 days or ICU mortality, suggesting that cisatracurium did not have a substantial impact on these critical outcomes.

Two retrospective studies using propensity-matched analyses investigated whether continuous infusions of cisatracurium were associated with better outcomes compared to vecuronium in patients with ARDS. Both studies reported no significant differences in mortality or hospital length of stay between the two groups [43,44]. However, the first study, which included 3,802 patients, found that cisatracurium was associated with fewer ventilator days and a shorter ICU length of stay [43]. In contrast, the second study, involving a smaller cohort of 58 patients, found no significant differences in these clinical outcomes [44]. The observed discrepancies may be attributed to the substantial difference in sample sizes between the two studies.

Two retrospective, observational cohort studies evaluated the clinical outcomes of ARDS patients treated with either cisatracurium or atracurium. The first study was a single-center analysis involving 76 patients with severe ARDS (PaO_2_/FiO_2_ ≤ 150) who received treatment within 72 hours of diagnosis [45]. The outcomes assessed included PaO_2_/FiO_2_ improvement at 72 hours, VFDs, ICU length of stay, hospital length of stay, and hospital mortality. No significant differences were observed between the cisatracurium and atracurium groups in any of these clinical outcomes. The second study was a multicenter analysis that included 225 ARDS patients (PaO_2_/FiO_2_ < 300 mmHg) to provide further investigations [46]. This study found a significant improvement in the PaO_2_/FiO_2_ ratio at 24 and 72 hours for patients receiving atracurium compared to cisatracurium. However, similar to the previous study, there were no significant differences in the duration of mechanical ventilation, ICU length of stay, or hospital length of stay. Notably, hospital mortality was significantly higher in the atracurium group, which could be attributed to unbalanced baseline characteristics, including a higher proportion of moderate to severe ARDS, older age, and a greater prevalence of COVID-19 diagnoses in this group. Despite these findings, both retrospective studies reported significant cost reductions with the use of atracurium compared to cisatracurium, suggesting that atracurium may be a safe and more cost-effective alternative for managing ARDS. Further studies are needed to address the limitations of these retrospective analyses.

## Beta-2 agonists

Beta-2 agonists are potential pharmacological agents for managing ARDS due to their modulation of key pulmonary cellular pathways involved in its pathophysiology. These agents reduce neutrophil sequestration, activation, and the production of inflammatory cytokines [48]. Additionally, they activate β-2 receptors on alveolar type-1 and type-2 cells, leading to increased intracellular cyclic adenosine monophosphate (cAMP) levels [49]. This activation enhances sodium transport, accelerates alveolar fluid reabsorption, and helps alleviate pulmonary edema in patients with ARDS [50].

To date, three randomized controlled trials have been conducted to evaluate the safety and therapeutic efficacy of both aerosolized and intravenous β2-agonists in the management of ARDS. These studies aimed to determine whether these agents could enhance critical patient-centered outcomes, such as reducing mortality rates or increasing VFDs. Unfortunately, none of the trials demonstrated a significant independent benefit in these key outcomes, highlighting the need for further research to explore alternative therapeutic strategies or refine the use of β2-agonists in this clinical context [14,15,16]. (Table 4)

**Table 4.** Beta-2 agonists in ARDS

**Reference**	**Study design**	**Number of patients**	**Beta-agonists type and dose**	**Key findings**
[14]	RCT	40 patients with ARDS Albuterol group (n=19) Placebo group (n=21)	IV albuterol infusions run at 0.075 ml/kg/h (15 μg/kg/h) for 7 days	↔ PaO_2_/FiO_2_ ratio at day 7 ↔ VFDs ↔ 28-day mortality ↔ Incidence of supraventricular arrhythmias
[15]	RCT	282 patients with ARDS Albuterol group (n=152) Placebo group (n=130)	Aerosolized albuterol (5 mg) q 4 hours for up to 10 days	↔ VFDs ↓ ICU free days in the albuterol group (13.5 vs 16.2; P=0.023) ↔ Mortality outcome at day 60 and at day 90 ↔ Organ failure free days ↔ Cardiac arrhythmias
[16]	RCT	326 patients with moderate to severe ARDS Albuterol group (n=162) Placebo group (n=164)	IV albuterol (15 μg/kg ideal bodyweight per hour) for up to 7 days	↑ 28-day mortality (34 vs 23%; P=0.03) ↓ VFDs (8.5 vs 11.1 days; P < 0.05) ↓ Organ failure free days (16.2 vs 18.5 days; P < 0.05) ↑ Incidence of cardiac arrhythmia (9 vs 2%; P < 0.05)

Randomized Clinical Trial (RCT); Partial pressure of oxygen in arterial blood (PaO_2_) to the fraction of inspiratory oxygen concentration (FiO_2_); Ventilator free days (VFDs); Intensive care unit (ICU); ↑=Increase; ↓= Decrease; ↔ = No significant change

The β-agonist lung injury trial (BALTI) was a single-center, double-blind, randomized controlled study that evaluated the impact of intravenous albuterol in patients with ARDS. The trial enrolled 40 mechanically ventilated patients within 48 hours of ARDS onset, randomly assigning them to receive either a sustained infusion of albuterol or a placebo for seven days. Results from the trial demonstrated that albuterol significantly reduced extravascular lung water after seven days of treatment, suggesting potential physiological benefits. However, no statistically significant differences were observed between the albuterol and placebo groups in key clinical outcomes, including the PaO_2_/FiO_2_ ratio, VFDs, or 28-day mortality rates. Additionally, patients in the albuterol group experienced a higher incidence of cardiac arrhythmias, raising concerns about its safety profile in ARDS population [14].

The Albuterol to Treat Acute Lung Injury (ALTA) trial was a multicenter, randomized clinical study that investigated the efficacy of aerosolized albuterol in improving outcomes for patients with ARDS. The trial enrolled 282 mechanically ventilated patients and aimed to evaluate whether administering aerosolized albuterol for 10 days could enhance clinical measures such as VFDs and reduce mortality compared to a saline placebo. The findings revealed no statistically significant differences between the albuterol and placebo groups in terms of VFDs, 60- and 90-day mortality rates, organ failure–free days, or the incidence of cardiac arrhythmias. Interestingly, the albuterol group demonstrated fewer ICU-free days compared to the placebo group, raising concerns about its potential impact on recovery. Due to the lack of observed benefit, the trial was terminated early for futility [15]. A secondary analysis of the ALTA trial revealed that the concomitant use of aerosolized albuterol and vasoactive agents in patients with ARDS was associated with a reduction in both VFDs and ICU-free days. This finding suggests the possibility of an uncharacterized interaction stemming from the additive effects of β-agonism in this critically ill population. Considering the widespread use of both agents, it is essential to prospectively assess the combined adverse effects associated with beta-agonism [51].

The β-Agonist Lung Injury Trial-2 (BALTI-2) was a multicenter, randomized clinical trial that investigated the effects of intravenous albuterol in patients with ARDS. The study enrolled 326 mechanically ventilated patients within 72 hours of ARDS onset, randomly assigning them to receive either intravenous albuterol or a placebo for seven days. The primary objective was to evaluate whether systemic albuterol could improve key clinical outcomes, such as VFDs and mortality. Contrary to expectations, the results revealed that systemic albuterol treatment significantly reduced VFDs and organ failure–free days. Additionally, it was associated with a higher 28-day mortality rate and an increased incidence of adverse cardiac events. Due to these concerning safety findings, the trial was terminated early [16].

## Conclusion and future perspectives

ARDS remains a complex and challenging condition to manage due to its heterogeneous nature and multifactorial causes. This review highlights the current pharmacological strategies, including corticosteroids, NMBAs, and beta-2 agonists, while underscoring their limitations in efficacy and safety. The variability in drug types, doses, routes of administration, and treatment timing points to an urgent need for standardized protocols tailored to specific ARDS subpopulations. One of the distinctive aspects of this review is its focus on addressing these gaps by highlighting the specific drug types from each class used in ARDS management as well as the targeted patient populations.

Despite the widespread use of corticosteroids and NMBAs, significant gaps persist in determining optimal drug regimens for different ARDS etiologies, such as sepsis-induced or COVID-19-associated ARDS. The observed differences in therapeutic responses—for instance, the preference for hydrocortisone in sepsis-related ARDS versus dexamethasone or methylprednisolone in COVID-19 ARDS—raise crucial questions about the possibility of etiology-specific treatment approaches. These unanswered questions necessitate further research to identify preferred agents and assess their efficacy across diverse patient cohorts.

The safety profile and cost-effectiveness of therapeutic agents also remain critical areas of exploration. While cisatracurium is favored among NMBAs for its safety profile, the cost advantages of alternatives like atracurium warrant additional comparative studies. Similarly, the systemic administration of beta-2 agonists like albuterol has been associated with unfavorable outcomes, highlighting the need for further evaluations of alternative delivery methods. In addition, investigations into pharmacological interactions, such as the potential adverse effects of combined beta-agonist and vasoactive agent therapies, are essential to improving treatment safety.

In conclusion, this review highlights recent evidence on the advantages and limitations of pharmacological treatments for ARDS, stressing the urgent need for further research to address the current challenges.
